# Assessing the antimicrobial activities of Ocins

**DOI:** 10.3389/fmicb.2015.01034

**Published:** 2015-09-28

**Authors:** Shilja Choyam, Dhanashree Lokesh, Bettadaiah Bheemakere Kempaiah, Rajagopal Kammara

**Affiliations:** ^1^Department of Protein Chemistry and Technology, Central Food Technological Research Institute (CFTRI)Mysore, India; ^2^Department of Spice and Flavor Science, Central Food Technological Research InstituteMysore, India

**Keywords:** bacteriocins, zone of inhibition (ZOI), diffusion, antimicrobial peptides, bifidobacteria, *Enterococcus*

## Abstract

The generation of a zone of inhibition on a solid substrate indicates the bioactivity of antimicrobial peptides such as bacteriocin and enterocin. The indicator strain plays a significant role in bacteriocin assays. Other characteristics of bacteriocins, such as their dispersal ability and the different zymogram components, also affect bacteriocin assays. However, universal well diffusion assays for antimicrobials, irrespective of their ability to diffuse (bacteriocin and enterocin), do not exist. The ability of different zymography components to generate non-specific activities have rarely been explored in the literature. The purpose of the present work was to evaluate the impact of major factors (diffusion and rate of diffusion) in a solid substrate bioassay, and to document the adverse effects of sodium dodecyl sulfate in zymograms used to estimate the approximate molecular weight of bacteriocins.

## Introduction

Bacteriocins/enterocins are produced by Gram-positive bacteria, and they exhibit narrow to broad spectrum antimicrobial activities that are heat stable, and bacteriocin/enterocin-producing strains are immune to the effects of these antibacterial peptides. Clusters of genes encoding bacteriocins are polycistronic, and they can be carried on chromosomes, plasmids, or transposons. The basic principle involved in the antimicrobial activity of bacteriocins is the destabilization of bacterial membranes. Certain standard and control strains are extensively used to detect the antimicrobial activities of bacteriocins. In most cases, *Micrococcus luteus* is the microbe used to assay bacteriocins antimicrobial activity in a plate assay. Recently, many more indicator strains, such as *Lactobacillus*, *Leuconostoc*, and *Pediococcus* strains, have been used in bacteriocin assays, although they are not widely available. The plate assay does not provide any information pertaining to the approximate molecular weights of bacteriocins. Therefore, a standard zymography procedure has been used to estimate the approximate molecular weights of bacteriocins for many years. However, recent observations in our laboratory demonstrate that zymograms are not suitable for determining the approximate molecular weights of antimicrobial proteins because of the lack of specificity due to a non-specific zone of growth suppression. The present manuscript reports various kinds of a non-specific zone of growth inhibition observed by following a zymogram approach.

Bacteriocins are antimicrobial peptides produced by *Lactobacillus* strains, and enterocins are antimicrobial peptides produced by *Enterococcus* strains. The potential uses of these antimicrobial peptides in the healthcare (Papagiani, 2003) and food industries has generated great interest in these industries, as well as in academia. This has led to basic and applied research on bacteriocin production and purification, as well as their applications. The antimicrobial peptide nisin, produced by some strains of *Lactococcus lactis*, is the most widely studied bacteriocin, and it has a generally recognized as safe (GRAS) status in the USA and in some countries in the EU, as is also the case for the antimicrobial peptide pediocin (Chikindas et al., 1995; Venema et al., 1995; Turcotte et al., 2004). A major difficulty in antimicrobial peptide research and applications is identifying them using bioassays. The growth inhibition produced by a microbe targeting another sensitive microorganism (Joosten and Nuñez, 1995; Wan et al., 1995; Rasch and Knochel, 1996; Turcotte et al., 2004), such as *M. luteus*, and *Leuconostoc* and *Pediococcus* strains, is often used in this regard.

Various methods and sensitive instruments, such as matrix-assisted laser desorption/ionization-time of flight mass spectroscopy, and microbiological agar plate assays, etc., are standard methods used to assay bacteriocins. Previously, the potency of bacteriocins has been determined using a variety of techniques that are based on either broth dilution or plate test methods. Later, newer methods have been produced based on the radial diffusion properties of bacteriocins, such as placing filter paper disks on the surface of the medium and cutting circular wells into the surface of gelatin plates. More sophisticated assays, such as the enzyme-linked immunosorbent assay (ELISA; Bouksaim et al., 1999), ATP bioluminometry (Waites and Ogden, 1987), radiometry (Culter et al., 1989), conductance measurements (Giraffa et al., 1990), and others, have been developed. They have not gained wide acceptance because they require technical expertise, dedicated equipment, and, moreover, the results of such (indirect) methods do not necessarily correlate with antimicrobial activity. Therefore, growth inhibition studies are the most commonly used methods in everyday trials. However, recent developments in our laboratory conclude that primary assays, such as agar diffusion (Tramer and Fowler, 1964; Rogers and Montville, 1991; Wolf and Gibbons, 1996) or disk diffusion assays, that are used for bacteriocins may not be suitable for enterocins. The major drawback of these assays is that they are influenced by the diffusibility of active substances such as bacteriocins and enterocins. Previously, diffusion/distribution-related problems were encountered and solved by employing the turbidometric assay (Isaacson and Kirschbaum, 1986; Nuñez et al., 1996; Cavo et al., 1999); however, this assay is very laborious and requires skilled personnel and had other essential requirements. Therefore, one needs a novel, efficient, accurate, and simple method for identifying bacteriocins and enterocins. Furthermore, to understand or estimate their approximate molecular weight, zymograms are not suitable because of their lack of specificity. Thus, keeping in mind all of the above observations, we developed novel and specific individual methods to assay bacteriocins and enterocins. We also show that zymograms are not sufficiently specific because they can result in the formation of a non-specific zone of growth inhibition, irrespective of the presence or absence of bacteriocins and enterocins.

Thus, the primary objective of the present work was to judge the suitability of the agar diffusion assay for the identification of bacteriocins. After identifying such antimicrobials, it is essential to calculate their approximate molecular weight by zymography. It is well understood that the rate of diffusion differs between bacteriocins/enterocins produced by the same species. Therefore, the same agar diffusion test may not be suitable for assaying both bacteriocins and enterocins. Using a standardized test for bacteriocins/enterocins could pose a severe problem if they do not exhibit any antimicrobial activity in the diffusion assay. Here, we have developed a novel agar diffusion assay that is suitable for identifying both bacteriocins and enterocins. Subsequently, their approximate molecular weights were estimated using conventional zymography that relied upon denaturing or native polyacrylamide gel electrophoresis (PAGE), whereas previous zymograms employed a basic reagent such as SDS, which may result in a lack of specificity by lysing the cells. Hence, it is essential to realize the importance of each reagent used in the zymogram to produce more effective methods for the estimation of the molecular weights of bacteriocins and enterocins. The present study describes the development of simple and universal agar diffusion methods for bacteriocin and enterocin assays. Similarly, we discuss the issue of using SDS in zymograms and the formation of a non-specific zone of growth suppression.

## Materials and Methods

### Stock Cultures of Test Organisms

Required and essential stock cultures of *M. luteus*, *Enterococcus gallinarum*, *Bifidobacterium catenulatum*, and *Lactococcus plantarum* (other strains as shown in the **Table 1**) were obtained from different sources, such as the Microbial Type Culture Collection (MTCC, Institute of Microbial Technology, An international Microbial, Fungal Repository, India; New England Biolabs, 240 County Road, Ipswich, MA 01938-2723, USA) and the German Culture Collection Center (DSM, Inhoffenstraße, 7B38124, Braunschweig, Germany). For long-term storage, stock cultures were kept at -80°C in 20% glycerol. For short-term storage, agar plates were stored at 4°C, and a new plate was streaked every 15 days. For a single subculture, the required microbes were grown either in de Man, Rogosa, and Sharpe (MRS) broth medium or Bifido broth. The indicator organism was *M. luteus* grown either in Luria-Bertani (LB) or MRS media (Hi-media laboratories, Mumbai, India) at 37°C, pH 6.5–7, for 24 h. During cultivation, optical density readings were performed at 600–700 nm.

**Table 1:** Strains used in this study.

Bacterial strain	Reference
*Aromonas hydrophila*	This study
*Bacillus subtilis*	MTCC
*Bacillus clausii*	This study
***Bifidobacterium catenulatum***	DSM
*Escherichia coli* (DH5a)	NEB
***Enterococcus gallinarum***	MTCC
***Lactococcus plantarum***	MTCC
*Listeria monocytogenes*	Stuart Peterson, 1940
*Micrococcus luteus*	MTCC, type strain
*Pseudomonas aureginosa*	MTCC, Rose, 1963
*Pseudomonas putida*	Type strain MTCC, ATCC 17440, DSM50188
*Salmonella typhi*	MTCC, Kauffman, 1941
*Streptococcus aureus*	This study
*Streptococcus aureginosa*	This study
*Streptococcus thermophillus*	MTCC, NCTC 10353
*Vibrio harvey*	This study
*Vibrio parahaemolyticus*	Allen and Baumann, 1971
*Vibrio mimicus*	This study
*Vibrio pluvialis*	This study

*Bold indicates the strains secreting antimicrobial agents. MTCC, Microbial Type Culture Collection Centre and Gene Bank, Institute of Microbial Technology, Sector-39A, Chandigarh-160036, India*.

### Growing and Harvesting of Antimicrobial Peptides (Bacteriocins/Enterocins)

Bifidobacterium, *Enterococcus*, and *Lactococcus* strains were (**Table 1**) streaked on freshly prepared MRS or brain-heart infusion (BHI) agar plates. After overnight incubation under anaerobic conditions at 37°C, a single isolated colony was inoculated into fresh MRS/BHI broth and grown for 12–16 h without shaking at 37°C. The resulting culture was subcultured (4–5%, vol/vol) overnight in fresh medium, followed by growth under the aforementioned conditions. After 4–5 h of growth, the culture was subjected to centrifugation for 10 min at 3,000 *g* to harvest the cells. The resulting supernatant was collected, and passed through a 0.2-μm filter (Millipore, Bangalore, India) and based on the necessity it was concentrated by using Millipore concentrators with a 5-kDa molecular weight cutoff. The resulting concentrated supernatant was subjected to a well diffusion, disk diffusion, or unique well diffusion assay (UWDA; our method).

### Agar Diffusion Assay (Well, Disk diffusion, and Unique Well Diffusion)

Agar (MRS/Bifido; 2%) was used for solid substrates. To prevent contamination, we decided not to use any surfactant, such as Tween-80 and Tween-20, as they increase the diffusion rate of the bacteriocin or enterocin on the agar (Wolf and Gibbons, 1996). Our preliminary tests for the enterocin assay showed no differences in activity, even after using Tween-20. However, bacteriocin activity was slightly increased by Tween-20. Therefore, bacteriocins, but not enterocins, respond positively toward to Tween 20. After tempering molten agar (fixed volume) at 40°C for 30 s, the indicator organism in its mid-exponential phase was added at a 1% concentration and mixed well. Subsequently, the agar was poured into sterile Petri plates in a sterile hood and allowed to solidify. Later, 5-mm diameter wells were constructed with a sterile aluminum bore maker (Hi-media Laboratories, Mumbai, India). Standard protocols were followed in terms of the amount and state of the indicator strain used in the experiment. Daba et al. (1991) used 50 μl of a 100-fold dilution of an overnight culture, while Mortvedt and Nes (1995) used approximately 20 μl of fresh indicator strains with an optical density at 600 nm (OD_600_) between 0.1 and 0.6. Briefly, in the present experiments we used 25–50 μl of an indicator strain at an OD_600_ of 0.2–0.6 for all the bacteriocin and enterocin assays. Many indicator strains as shown in the **Table 1** were used for the study. Bacteriocin or enterocin (50–100 μl) was pipetted into each well, and the plates were pre-incubated at room temperature for 30 min to increase the absorbance of the antimicrobials.

**FIGURE 1:**
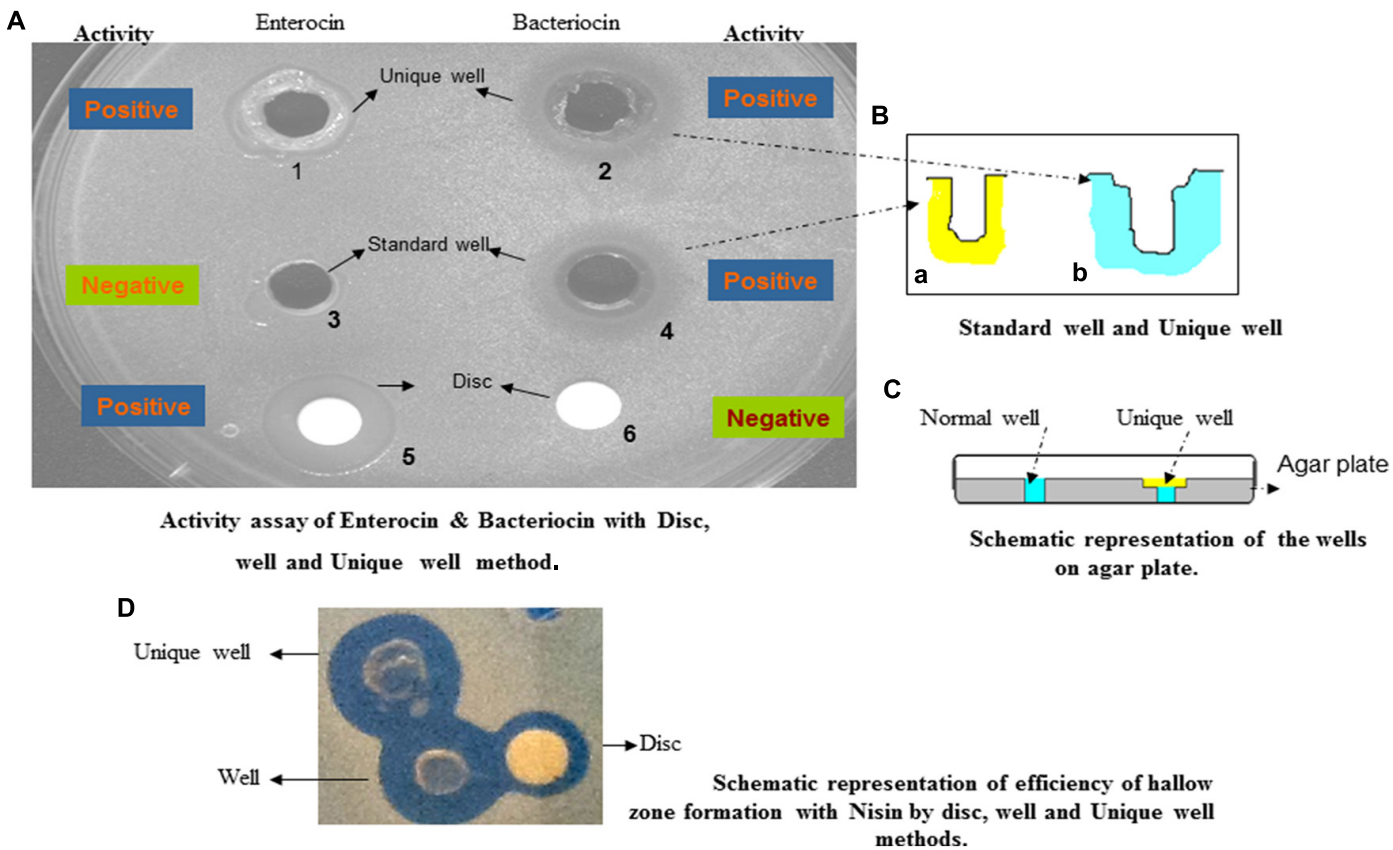
**(A–D)** Bacteriocin and enterocin inhibition of a *Micrococcus luteus* indicator lawn on de Man, Rogosa, and Sharpe (MRS) agar medium. The producer strains are *Bifiodobacterium catenulatum* and *Enteroccus gallinarum.* **(A)** Wells 1 and 2 are unique wells, and they share the same structure as shown in **(B)**. Wells 3 and 4 are conventional wells made by a gel borer, and they share the same structure as shown in **(B)**. Numbers 5 and 6 are sterile disks placed on the lawn. Wells 1 and 3 and disk 5 correspond to enterocin, and wells 2 and 4, and disk 6 correspond to bacteriocin. **(D)** Nisin subjected to three different kinds of assays.

The standardized test was repeated without making any wells on the plate, but by placing sterile disks (Hi-Media laboratories, Mumbai, India; 5–10 mm diameter) on the agar surface (**Figure 1A**, wells 5 and 6). The bacteriocin/enterocin-saturated disks were used in the well diffusion assays (**Figure 1A**, wells 1 and 3; **Figure 1B** well a; and **Figure 1C**) after drying the plates for 30 min to allow the absorption and distribution of the bacteriocin into the medium. Eventually, the plates were dried and then incubated at 37°C for 24 h or until ZOI developed to compare the bacteriocin and enterocin activities under different conditions. The diameters of the ZOI in the assay plates were measured using images of the plates and graded scales. The tests were performed in triplicate.

A unique and simpler assay method was also followed for enterocins, as the agar well diffusion assay was suitable. Here, wells were constructed in such a manner that the addition of bacteriocin or enterocin leads to the total exposure of the agarose surface to the cells (**Figure 1A**, wells 2 and 4; **Figure 1B**, well b; and **Figure 1C**). This novel process further helps in distributing the bacteriocin/enterocin in close proximity of the cell surface, even if they had limited diffusibility. Subsequently, a similar process of bacteriocin/enterocin addition, followed by incubation and observation of cell lysis, was followed.

### Zymography

We performed zymography under different conditions, such as using samples with or without SDS sample buffer, native PAGE, denaturing PAGE, sample buffer with media, only sample buffer, only SDS; empty lanes were also run, to calculate the approximate molecular weights of bacteriocin and enterocins. Bifidobacterial or enterococcal samples were lysed with SDS sample buffer, boiled, and centrifuged at 9,000 *g* for 20 min. The supernatant was collected, and protein bands were separated on 10% SDS-PAGE. Subsequently, the gel was washed with sterile distilled water and overlaid on an MRS/Bifido agar plate containing the *M. luteus* indicator strain. Caution was used to prevent the formation of air bubbles between the gel and the assay plate. Along with the bacteriocins and enterocins, we also included positive and negative controls for SDS-PAGE gel, such as SDS alone, SDS sample buffer, media alone, etc. The overlay plate was incubated overnight at 37°C. Observations were recorded on the following day. The same samples were also graded using a native PAGE gel to determine the effect of each ingredient on cell growth inhibition. All these reagents were further subjected to the agar well diffusion assay to assay for growth inhibition.

### Establishment of a UWDA

A few novel chemical compounds with different molecular weights were considered for validating the assay.

### Synthesis of Zerumbone Oxime Esters

**FIGURE 2:**
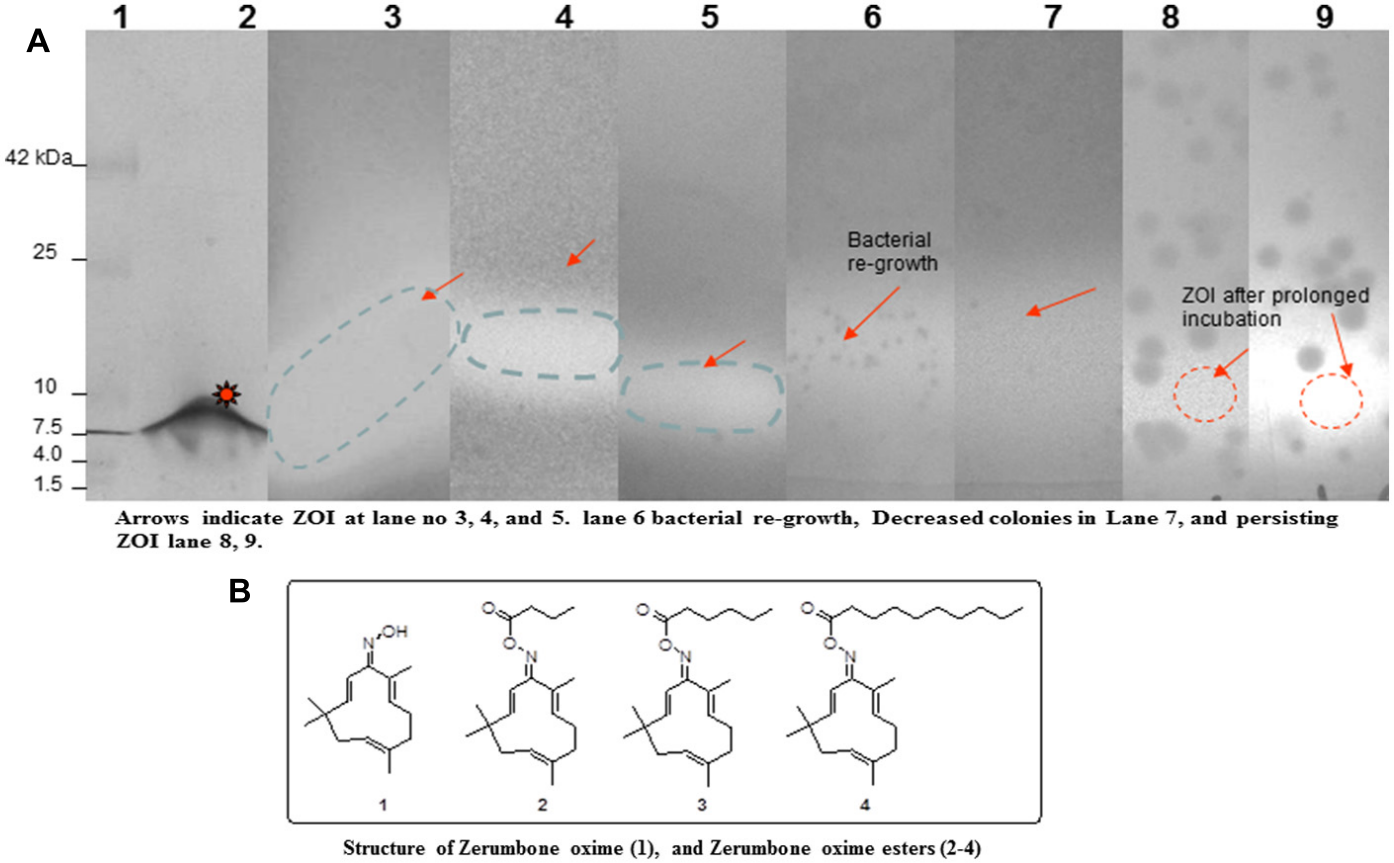
**(A)** Zymography to show the formation and disappearance of the non-specific zone of inhibition (ZOI). The procedure in brief, After the successful separation of proteins by SDS-PAGE, and native PAGE the gel was rinsed twice with phosphate buffer. Later, it was overlaid on indicator strain containing plate. Left overnight at 37°C. The sequence shown on the gel are: lane 1, molecular weight marker; lane 2, enterocin; lanes 3–5, overlaid gel showing the ZOI, and decreased ZOI; lane 6, Reappearance of bacterial colonies; and lanes 7–9, existing ZOI after prolonged incubation. **(B)** Structure of zerumbone oxime and its oxime-esters. Star represents for antimicrobial peptide.

In a 100-ml two-neck round-bottom flask, zerumbone oxime (1 mM) was added to 20 ml of CH_2_Cl_2_, 1-Ethyl-3-(3-dimethylaminopropyl) carbodiimide (EDCI, 2.5 mM), and 4-(Dimethylamino)pyridine (DMAP, 0.2 mM). Magnetic stirring was performed under a nitrogen atmosphere for 5 min to mix them efficiently. Subsequently, carboxylic acid (1 mM) was added, and the resultant reaction mixture was stirred at room temperature until the reaction was completed. The progress of the reaction was monitored by thin-layer chromatography using ethyl acetate and the hexane mixture as an eluting solvent for the disappearance of zerumbone oxime. After the reaction was completed, it was diluted with 40 ml water. The organic layer was separated, dried over anhydrous Na_2_SO_4_, and filtered. The clear filtrate was concentrated to yield a crude product, which was purified by triturating with petroleum ether. Pure compounds (2–4) were characterized by nuclear magnetic resonance, infrared, and high-resolution mass spectroscopy. The structures of zerumbone oxime and its oxime esters have been shown in the **Figure 2B**.

## Results and Discussion

The diameters of the inhibition zones produced on plates in the agar diffusion assays for bacteriocin and enterocin were evaluated and analyzed using a graded scale. The analysis of variance for the agar diffusion test is shown in **Figure 1A**. It has been speculated that enterocins require a different indicator strain than *M. luteus*. Thus, during the screening, we successfully found a suitable *Bacillus subtilis* indicator strain for which enterocin showed a real inhibition zone. It was observed that *M. luteus* is the best indicator strain for bacteriocin. However, during the procedure, we noted that enterocin was not able to show any inhibition zone in the agarose well diffusion assay regardless of the indicator strain used, whereas bacteriocin was able to produce an inhibition zone regardless of the indicator strain used **Figure 1A**. To understand this phenomenon, we followed a new approach where two different wells were made in the agarose: one with a normal, well and the other with a unique well (**Figure 1**, wells 3 and 4, and wells 1 and 2). The loaded enterocin easily spread onto the surface of the agarose allowing enterocin to come into close proximity to the indicator strain’s surface. We also used the disk diffusion assay to find the optimal and universal conditions to simultaneously test for bacteriocins/enterocins.

We observed the formation of an inhibition zone when bacteriocin was subjected to the conventional agarose well diffusion assay, as well as to the unique well pattern discussed above. However, no inhibition zone was formed by a similar bacteriocin (which was used for **Figure 1A**, wells 2 and 4) in the disk diffusion assay. The effects were opposite in the case of enterocin, where the inhibition zone formation was not observed in the conventional agarose well diffusion assay (**Figure 1A**, well 3). However, an inhibition zone was easily identified in the UWDA (**Figure 1A**, well 1), similar to the case with the disc diffusion assay (**Figure 1A**, well 5). These results are illustrated in **Figure 1A**, wells 1 and 2, which clearly depicts that the novel well diffusion assay is suitable and universally applicable for both bacteriocins and enterocins. In contrast, the conventional agarose well diffusion assay cannot be used for growth inhibition experiments for bacteriocins and enterocins. Thus, we conclude that the unique well diffusion method is universally applicable for growth inhibition studies. The structure of the conventional wells and unique wells are depicted in **Figure 1B**.

### Estimation of the Approximate Molecular Weight of Bacteriocins/Enterocins

In a zymogram, there is a conventional SDS-PAGE separation of bacteriocin/enterocin, followed by overlaying the gel on an agar plate containing indicator cells (either *M. luteus*, *E. gallinarum and L. plantarum*, respectively) as shown in the **Table 1**. This method can be used to determine the growth inhibition zone, indicating bacteriocins/enterocins, and one can also estimate their approximate molecular weights, as the inhibition zone forms only wherever the protein is active. We observed a zone of growth inhibition by performing zymography not only for bacteriocins/enterocins, but also for lanes loaded with media, SDS sample buffer, SDS alone, and even empty wells prior to performing denaturing SDS-PAGE (**Figure 2A**). Identical results were observed in native PAGE. Even the SDS- and SDS sample buffer-containing wells showed a zone of inhibition (ZOI) in parallel with the wells loaded with enterocins/bacteriocins. We also observed an area of inhibition in native gels whose wells were loaded with either SDS or SDS sample buffer (**Figure 2A**). A ZOI was even found without loading any samples and by running the gels in the presence of running buffer containing SDS. **Figure 2** and **Table 2** clearly indicate the ZOI in native and SDS-PAGE.

**Table 2:** Formation of non-specific ZOI with different sample types in native and SDS-PAGE.

Native/SDS	Type of sample buffer	Type of running buffer	Type of sample/results						
			a	b	c	d	e	f	g
Native Page	Native	Native	Y	N	Y	N	Y	N	Y
	SDS	SDS	Y	Y	Y	Y	Y	Y	Y
	Native	SDS	Y	Y	Y	Y	Y	Y	Y
	SDS	Native	Y	Y	Y	Y	Y	Y	Y
SDS PAGE	Native	SDS	Y	Y	Y	Y	Y	Y	Y
	SDS	Native	Y	Y	Y	Y	Y	Y	Y
	Native	Native	Y	Y	Y	Y	Y	Y	Y
	SDS	SDS	Y	Y	Y	Y	Y	Y	Y

*a, only SDS sample buffer; b, empty wells; c, SDS only; d, native buffer; e, bifido broth; f, bacteriocin sample;g, denatured bacteriocin sample. Y indicates for Formation of ZOI and N for no ZOI*.

Amazingly, after incubation of these zymograms for a longer period at room temperature, we observed the growth initiation of the indicator strains. The lanes loaded with SDS sample buffer and media on native and denaturing gels resulted in a ZOI. However, no growth initiation was observed in the lanes corresponding to enterocin/bacteriocin-loaded wells (**Figure 2A** arrows indicate). Thus, the area of inhibition in these lanes was larger and permanent as no development was started later. The area of inhibition that formed in the initial stages was transient. Afterwards, it was observed that as the time of incubation increases, the ZOI decreases, and bacterial growth were also found in the same region, where previously there was an area of inhibition.

## Conclusion

In this study, we showed that the conventional agarose well diffusion assay is not suitable and applicable for enterocins, but are well suited for assaying bacteriocins. Likewise, the disk diffusion method is highly suitable and appropriate for enterocins, but not for bacteriocins. Thus, we successfully developed a unique, simple, and universal method for identifying enterocins/bacteriocins that form a ZOI by developing a unique type of well. We call this the UWDA for bacteriocins/enterocins/lactocins. Furthermore, the results of this study confirm that the UWDA is a universal method that is applicable for any kind of bacteriocin/enterocin/lactocin, regardless of their diffusibility. Conventional well-diffusion tests are considered to produce confounding results, as well as false-negatives if novel bacteriocins/enterocins/lactocins require unique conditions. Therefore, we suggest that the UWDA method be used for the identification of bacteriocins/enterocins/lactocins, as it is reproducible and highly efficient.

The method of estimating the approximate molecular weight by zymography has significant drawbacks. Among them, the formation of ZOI by SDS because of its detergent property results in cell lysis. Thus, samples containing trace amounts of SDS in the gel used for a zymogram can create false and non-specific ZOI. Thus, the use of trace amounts of SDS in zymograms should be avoided. One should preferably use native PAGE, and it should not contain even a trace amount of SDS, even in the running buffer. In the end, we conclude that the UWDA is universally applicable for enterocin or bacteriocin bioassays. Trace amounts of SDS in the system will create non-specific ZOI and, thus, should be avoided.

To determine whether the ZOI formed on zymograms were artifacts or truly resulted from bacteriocin/enterocin/lactocin activity, the SDS-overlaid plates were allowed to incubate for 15–16 h at room temperature. It was shown that wherever there was a non-specific ZOI the bacterial growth was observed (gel lanes loaded with SDS, sample buffer, SDS running buffer, etc.; **Figure 2A**, lanes 3, 4, and 6). However, along the surface of real ZOI, i.e., inhibition due to bacteriocins/enterocins, no bacterial growth was observed (**Figure 2A**, lane 3). Therefore, we conclude that bacteria can grow in the non-specific ZOI following prolonged incubation, but they cannot grow in actual ZOI due to the activities of bacteriocins or enterocins (**Figure 2A**, lanes 5, 7, and 8), which are stable. Thus, it is concluded that SDS also forms ZOI in the bacteriocin assay, but such zones are temporary/transient and only persist for 12 h (**Table 2**). Therefore, further incubation of the plates is necessary to understand the ZOI status. However, the ZOI created by either bacteriocins or enterocins were static since further incubation of the plates that were overlaid with SDS did not result in the degradation of the ZOI (**Figure 2A**, lane 9).

Hence, we suggest that zymography should not be used to determine the approximate molecular weights of bacteriocins/enterocins, but merely to determine that the ZOI resulted from bacteriocin/enterocin activity. It is indeed indispensable to avoid the use of SDS when running gels, and the prolonged incubation of overlaid plates is also required. In the end, we conclude that zymograms are the proper choice for estimating the approximate molecular weight of enterocins/bacteriocins, provided that the aforementioned recommendations are adopted.

### Validation of the UWDA

Compounds 1–4 were synthesized from zerumbone, a sesquiterpene isolated from wild ginger (Z*ingiber zerumbet*). Zerumbone oxime (compound 1) was prepared by reacting zerumbone with hydroxylamine hydrochloride. Compounds 2–4 are zerumbone oxime esters obtained by the reaction of compound 1 (**Figure 2B**) with alkanoic acids, such as butyric, hexanoic, and decanoic acid. Compounds 2–4 and other long chain fatty acid esters of zerumbone, together called ZOFA’s we’re prepared to study their antibacterial and antimutagenic potential. In the present study, only a few ZOFA’s were selected based on their polarity (**Figure 2B**). The polarity of compounds 1–4 decreases as the length of the lipophilic alkyl side chain attached to the oxime increases. Compound 1 is, more often than not, polar in nature, while compound 4 is least polar among the compounds selected and compounds 2 and 3 have polarity in between compounds 1 and 4. The oxime functionality is present in compound 1, whereas the oxime-ester linker was common among compounds 2–4.

Synthesized zerumbone oxime (compound 1) and zerumbone oxime esters (compounds 2–4) were subjected to the UWDA. Prior to subjecting them to the UWDA, they were assessed for their antimicrobial function via the well and disk diffusion assays. As the side chain length increased from 1–4, the antimicrobial activity was reduced, or negligible activity was observed. Subsequently, all of them were subjected to the UWDA, and the results showed that as their molecular weight increased, their diffusion rate decreased, thereby affecting their natural activity. Consequently, the activity was much higher in the case of zerumbone oxime, followed by derivatives 2–4. There was absolutely no activity in the case of zerumbone oxime derivative 4, indicating that the lack of the diffusion ultimately affected its activity. However, the same compounds were observed to contain reproducible and efficient activity when subjected to an ELISA-based antimicrobial assay (data not shown). The validation and comparison of the well diffusion, disk diffusion and UWDA with different indicator strains and their efficiency of formation of ZOI shown in **Table 3**.

**Table 3:** Comparative study of bacteriocin, lactocin, and enterocin on different formats such, as well diffusion (WD), unique well diffusion (UWD), and disk diffusion (D).

S.NO	Bacterial strain	Bacteriocin			Enterocin			Lactocin		
		WD	UWD	D	WD	UWD	D	WD	UWD	D
1	*Escherichia coli*	-	+	-	-	+	P	-	+	P
2	*Bacillus subtilis*	-	+	P	-	+	P	-	+	P
3	*Pseudomonas Aeriginosa*	-	+	-	-	+	-	P	+	P
4	*Streptococcus thermophilus*	-	P	P	-	-	-	P	+	P
5	*Pseudomonas putida*	-	+	P	-	+	P	-	+	P
6	*Micrococcus luteus*	+	+	P	-	+	P	-	P	P
7	*Bacillus clausii*	-	+	P	-	+	P	-	+	P

*P represents Normal*.

## Author Contributions

SC and DL executed the idea; BBK validated the experimental data, and KR conceptualized, planned, designed, analyzed, Interpreted results, and wrote the manuscript.

## Conflict of Interest Statement

The authors declare that the research was conducted in the absence of any commercial or financial relationships that could be construed as a potential conflict of interest.
